# Supplementary material for: Public Perceptions on the Policy of Electronic Cigarettes as Medical Products on Twitter
Source: Int J Environ Res Public Health. 2023 Feb 1;20(3):2618. doi: 10.3390/ijerph20032618 (PMC9915110; doi:10.3390/ijerph20032618)
Supplement: Supplementary file 1 [file ijerph-20-02618-s001.zip › ijerph-2128356-supplementary.pdf]

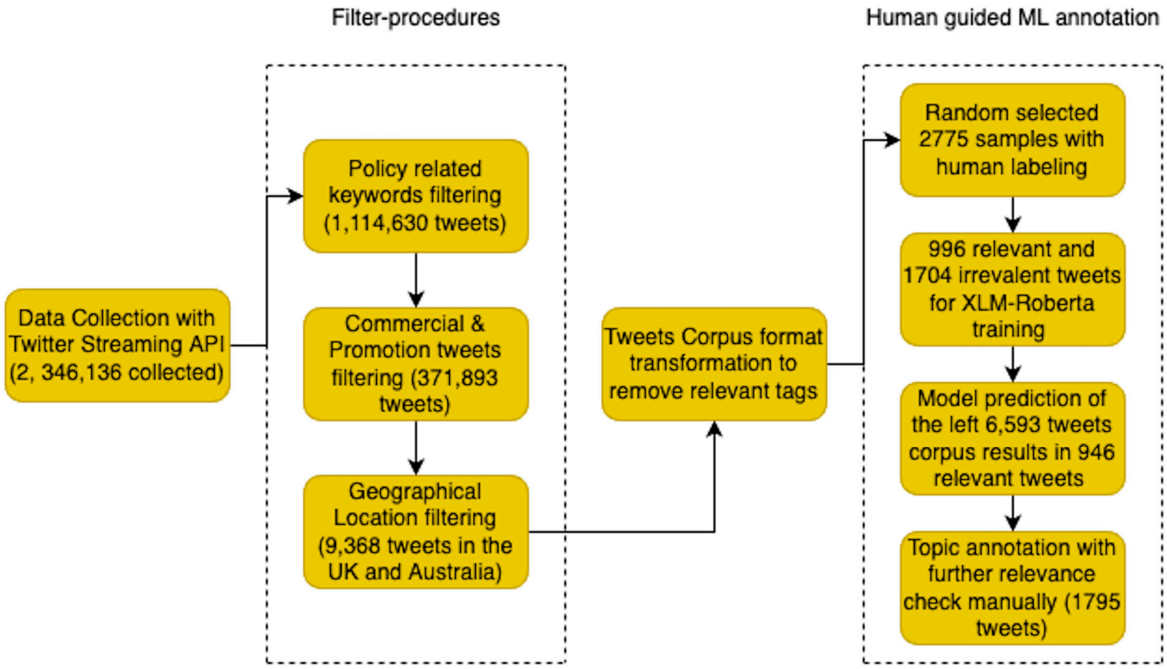

Figure S1. The diagram of data preprocessing procedures.

Table S1. Topics in tweets related to e-cigarettes as medical products.

Attitude	Topics	Australia	UK	Example tweet
Positive	Health effect of e-cigarettes	57 (47.90%)	22 (16.06%)	"Dangerous and prohibited ingredients were found in two out of every three nicotine vaping products tested," @TGA and @ACTHealth say after joint operation. https://t.co/bd6P7teRyn (accessed on 3 December 2022)
	Effect on youth	25 (21.00%)	9 (6.57%)	New vaping laws a start in confronting the nicotine pandemic poisoning our children https://t.co/D7CsHId8qd (accessed on 3 December 2022) via @AMA
	Help quit smoking	18 (15.13%)	90 (65.69%)	NHS to prescribe e-cigarettes to help smokers kick habit<br><br>https://t.co/dM9ScV0fmD (accessed on 3 December 2022)
	Professional instruction	15 (12.61%)	14 (10.22%)	"Having e-cigarettes available on prescription means that they'll come with the advice and support that smokers need from medical professionals.<br><br>"They'll also be available at higher strengths than consumer regulations allow, which will benefit more heavily-addicted smokers."

Attitude	Topics	Australia	UK	Example tweet
	Other reasons	14 (11.76%)	17 (12.41%)	In a recent article, Dr Califf detailed a âtrifectaâ of sweeping regulations: lowering the amount of nicotine in tobacco products to subaddictive levels; banning over-the-counter vaping products; and supporting prescription vaping so tobacco users donât go through withdrawals.
Negative	Prevent smoking cessation	147 (26.92%)	14 (4.58%)	disposables are awful yes, and are what seemingly causes most health problems + kids start on them and they have insane amounts of nic. but a lot of people with vape devices are ex smokers/smokers trying to reduce harm. this is just gonna cause so many people to go back to cigs.
	Hard to long-time vapers	55 (10.07%)	5 (1.63%)	It's ridiculously hypocritical and a massive windfall for doctors treating lung cancer. I'm not sure how that's not a serious conflict of interest. The bottom line is if people with a nicotine addiction can't vape they will smoke tobacco, and they will die at a massive expense.
	GPs' misinformation	56 (10.26%)	13 (4.25%)	Many Doctors have been exposed to poor guidance from @RACGP & @ama_media, making various unworkable

Attitude	Topics	Australia	UK	Example tweet
				recommendations that quite frankly would have a lot of Doctors checking their insurance policies.<br>Most doctors I have spoken to still don't even understand how #vaping works!
	Emotional Catharsis	89 (16.30%)	39 (12.75%)	@TGAgovau This is a ridiculous law. Vaping should be as free as smoking.
	The policy is not useful	54 (9.89%)	24 (7.84%)	Can anyone explain to me why I can use cigarettes with nicotine, nicotine gum, nicotine lollies or a nicotine inhaler but I am a criminal if I vape nicotine (without prescription). A person can still vape without nicotine so this new law does nothing to stop kids vaping. #vape
	Economic effect	72 (13.19%)	174 (56.86%)	E-cigs on the NHS? if smokers want to quit, they'll quit, vapes aren't that expensive, much cheaper than cigarettes. Instead of spending millions on that, use it for the CBD meds that are costing people 1000s a month yet changes their lives completely. (And I'm a smoker ð)
	Personal choice	14 (2.56%)	18 (5.88%)	@BGL66 @AngelComrade @TGAgovau No, it is not good. Whatever your or his opinion of vaping he has no right to impose

Attitude	Topics	Australia	UK	Example tweet
				it on anyone just because he is a member of a gatekeeping priesthood. Aldults have the right to make their own choices, even if those choices entail some unknown, purported risk.
	Other regulatory needs	67 (12.27%)	5 (1.63%)	Banning vapes but not cigarettes imagine my shock
	Other reasons	68 (12.45%)	47 (15.36%)	@talkRADIO @JuliaHB1 @DeborahArnott They might prescribe e-cigs for this who donât smoke to protect those who do..! ð gov strategy moving forwardâ¦
